# Adverse Events of Anti-Tumor Necrosis Factor α Therapy in Ankylosing Spondylitis

**DOI:** 10.1371/journal.pone.0119897

**Published:** 2015-03-12

**Authors:** Qiang Tong, Qing Cai, Tristan de Mooij, Xia Xu, Shengming Dai, Wenchun Qu, Dongbao Zhao

**Affiliations:** 1 Department of Rheumatology and Immunology, Changhai Hospital Second Military Medical University, Yangpu District, No.168 Changhai Road, Shanghai-200433, China; 2 Department of Neurosurgery, Mayo Clinic, Rochester Minnesota, 55902, United States of America; 3 Dep. of Physical Medicine & Rehabilitation, Mayo Clinic, Rochester, Minnesota, 55902, United States of America; 4 Department of Anesthesiology Pain Division, Mayo Clinic, Rochester, Minnesota, United States of America; University of East London, UNITED KINGDOM

## Abstract

**Objective:**

This study aims to investigate the prevalence of short-term and long-term adverse events associated with tumor necrosis factor-α (TNF-α) blocker treatment in Chinese Han patients suffering from ankylosing spondylitis (AS).

**Methods:**

The study included 402 Chinese Han AS patients treated with TNF-α blockers. Baseline data was collected. All patients were monitored for adverse events 2 hours following administration. Long-term treatment was evaluated at 8, 12, 52 and 104 weeks follow-up for 172 patients treated with TNF-α blockers.

**Results:**

Short-term adverse events occurred in 20.15% (81/402), including rash (3.5%; 14/402), pruritus (1.2%; 5/402), nausea (2.2%; 9/402), headache (0.7%; 3/402), skin allergies (4.0%; 16/402), fever (0.5%; 2/402), palpitations (3.0%; 12/402), dyspnea (0.5%; 2/402), chest pain (0.2%; 2/402), abdominal pain (1.0%; 4/402), hypertension (2.2%; 9/402), papilledema (0.5%; 2/402), laryngeal edema (0.2%; 1/402) and premature ventricular contraction (0.2%; 1/402). Long-term adverse events occurred in 59 (34.3%; 59/172) patients, including pneumonia (7.6%; 13/172), urinary tract infections (9.9%; 17/172), otitis media (4.7%; 8/172), tuberculosis (3.5%; 17/172), abscess (1.2%; 2/172), oral candidiasis (0.6%; 1/172), elevation of transaminase (1.7%; 3/172), anemia (1.2%; 2/172), hematuresis (0.6%; 1/172), constipation (2.3%; 4/172), weight loss (0.6%; 1/172), exfoliative dermatitis (0.6%; 1/172). CRP, ESR and disease duration were found to be associated with an increased risk of immediate and long-term adverse events (P<0.05). Long-term treatment with Infliximab was associated with more adverse events than rhTNFR-Fc (P<0.01).

**Conclusion:**

This study reports on the prevalence of adverse events in short-term and long-term treatment with TNF-α blocker monotherapy in Chinese Han AS patients. Duration of disease, erythrocyte sedimentation rate, and c-reactive protein serum levels were found to be associated with increased adverse events with anti-TNF-α therapy. Long-term treatment with Infliximab was associated with more adverse events than rhTNFR-Fc.

## Introduction

Ankylosing spondylitis (AS) is a chronic inflammatory arthritis predominantly involving the axial spine and sacro-iliac joints. AS primarily manifests as pain, stiffness and progressive joint ankyloses, caused by underlying inflammatory processes [1–2]. While non-steroidal anti-inflammatory drugs (NSAIDs) are still considered the first line of treatment, concerns are raised that prolonged exposure may increase the rate of side effects [3]. The efficacy of disease-modifying anti-rheumatic drugs (DMARDs) is questionable, as they have not shown to prevent or reduce radiologically evident disease progression [4]. Tumor necrosis factor-alpha (TNF-α) plays a key role in the pathogenesis of many chronic inflammatory and rheumatic diseases, including AS. Randomized controlled trials of Etanercept and Infliximab, both TNF-α antagonists, have independently shown to delay disease progression and significantly reduce symptoms, thus improving both function and quality of life [5]. Considering the efficacy, safety and generally more favorable side-effect profile of TNF-α blockers, they are increasingly used as first-line treatment [6–7].

In China, anti-TNF-α drugs approved for medication include rhTNFR-Fc, Infliximab, Etanercept and Adalimumab. While the mechanism of action of these medications is similar, important differences do exist. rhTNFR-Fc (recombinant human Tumor Necrosis Factor-α Receptor II: IgG Fc Fusion Protein) constitutes a soluble variant of Etanercept [8]. Infliximab and adalimumab are both anti-TNF-α monoclonal antibodies, but whereas adalimumab is fully humanized, infliximab consists for 25% of murine peptides, possibly contributing to acute infusion reactions associated with this drug [9].

As anti-TNF therapy targets one of the central regulators of the inflammatory response, patients can be left vulnerable to infusion reactions, rashes, fever and papilledema [10,11]. Subtle differences in signal processing of the inflammation signaling cascade may exist between different races, causing differences in outcomes and complication tendencies [12]. Evaluating short-term and long-term adverse events associated with anti-TNF-α mono-therapy may thus provide critical information to optimize treatment both for the general patient population and this subgroup in particular. We studied the safety of rhTNFR-Fc and Infliximab mono-therapy in Chinese Han patients treated at our institution (Chang Hai hospital, Shanghai, China) by evaluating the occurrences of short-term and long-term adverse events.

## Patients and Methods

### Study design and patients

We conducted a prospective study aimed to evaluate the prevalence and severity of adverse events in AS patients treated with rhTNFR-Fc and Infliximab. Patients receiving mono-therapy treatment from June 2008 to February 2013 at the Department of Rheumatology of Changhai Hospital were eligible for enrollment in the study. We used the Modified New York Criteria (1984) for AS as inclusion criteria [13]. Exclusion criteria included past medical history of chronic infectious diseases, neoplasm, hepatic or renal dysfunction, hematological or cardiac conditions, or multiple sclerosis. Patients receiving DMARD co-medication were additionally excluded. Approval for the study was received from the Institutional Review Board of Changhai Hospital, affiliated to the Second Military Medical University, and written informed consent was obtained from all participants.

Patients received intravenous (IV) infusion of Infliximab and subcutaneous injections of rhTNFR-Fc according to our standard treatment protocols (Infliximab, 200mg IV at 0, 2 and 6 weeks, followed by every 6 weeks thereafter; rhTNFR-Fc, 25mg twice weekly subcutaneous injection). Patients were monitored for adverse events during two hours after administration of TNF-α blockers. Short-term adverse events were stratified into three grades (mild, moderate, severe). Mild adverse events were defined as complications spontaneously resolving within one hour, moderate adverse events were defined as complications that may be alleviated by medical interventions and severe adverse events were defined as fatal or requiring inpatient hospitalization. This is in parallel with Common Terminology Criteria for Adverse Events (CTC) [14].

### Statistical analysis

Patient characteristics and quantitative measures are presented as mean ± standard deviation. Statistical analysis was conducted using the Pearson’s chi square test or student’s t-test, where appropriate. Data was analyzed using SPSS version 17.0 (SPSS Munich, Germany). Results were considered statistically significant for p ≤ 0.05.

## Results

### Short-term adverse events

The study included 402 patients (375 men and 27 women) aged 39.63 ± 15.82 years. Men accounted for 93.3% of our cohort and averaged 39.63 ± 15.82 years of age. Infliximab mono-therapy was administered to 192 patients, while 210 patients received rhTNFR-Fc mono-therapy. See Table 1.

**Table 1:** Baseline characteristics of patients experiencing short-term adverse events.

	All patients	Non-adverse event patients	Adverse event patients	P-value
Demographics	(n = 402)	(n = 321)	(n = 81)	
Age (years)	39.6 ± 15.8	42.1 ± 19.0	35.9 ± 17.2	0.075
Men (%)	93.3%	92.2%	97.5%	0.087
Duration (years)	7.6 ± 8.2	10.1 ± 9.8	6.3 ± 7.6	0.001
Type				
Axial phenotype (%)	69.2%	75.1%	45.7%	1.02E-07
Biologic assessment				
HLA-B27, no (%)	95.5%	98.8%	82.7%	1.76E-09
Serum CRP (mg/dl)	36.4 ± 18.5	41.5 ± 17.4	31.3 ± 22.4	1.11E-05
ESR (mg/dl)	32.2 ± 15.3	38.0 ± 16.5	29.2 ± 15.3	1.95E-05
Drug treatment				
Infliximab (%)	47.8% (192)	48.9% (157)	43.2% (35)	0.3852
rhTNFR-Fc (%)	52.2% (210)	51.1% (164)	56.8% (46)	

CRP = C-reactive protein. ESR = erythrocyte sedimentation rate. Axial phenotype is ankylosing spondylitis which initially predominantly affects the spine and pelvic joints.

Short-term adverse events (arising within 2 hours of administration) occurred in 81 patients (20.2%), including 46 patients (11.4%) treated with rhTNFR-Fc and 35 patients (8.7%) treated with Infliximab. See Table 2. The total number of short-term adverse events did not differ significantly between treatment groups. Patients who experienced adverse events were on average younger than those remaining free of adverse events (35.9 ± 17.2 versus 42.1 ± 19.0 years, p = 0.075). The average disease duration was shorter in patients experiencing adverse events (6.3 ± 7.6 versus 10.1 ± 9.8 years, p = 0.001). Adverse events predominantly occurred in patients with primary disease symptoms to the peripheral joints (p = 10^-7^). Paradoxically, c-reactive protein level and the erythrocyte sedimentation rate were significantly higher in the unaffected group (p = 10^-5^).

**Table 2:** Comparison of short-term adverse events.

	All adverse event patients	rhTNFR-Fc	Infliximab	P-value
Mild (%)	(n = 81)	(n = 46)	(n = 35)	
rash	14 (17.3%)	11 (23.9%)	3 (8.6%)	0.0704
pruritus	5 (6.2%)	2 (4.4%)	3 (8.6%)	0.4339
nausea	9 (11.1%)	4 (8.7%)	5 (14.3%)	0.4277
headache	3 (3.7%)	1 (2.2%)	2 (5.7%)	0.4032
Moderate (%)				
skin allergies	16 (19.8%)	12 (26.1%)	4 (11.4%)	0.1007
fever	2 (2.5%)	2 (4.6%)	0 (0.00%)	0.2116
palpitations	12 (14.8%)	8 (17.4%)	4 (11.4%)	0.4542
dyspnea	2 (2.5%)	1 (2.2%)	1 (2.9%)	0.8443
chest pain	1 (1.2%)	0 (0.0%)	1 (2.9%)	0.2486
abdominal pain	4 (4.9%)	1 (2.2%)	3 (8.6%)	0.1880
hypertension	9 (11.1%)	2 (4.4%)	7 (20.0%)	0.0263
Severe (%)				
papilledema	2 (2.5%)	2 (4.4%)	0 (0.0%)	0.2116
laryngeal edema	1 (1.2%)	0 (0.0%)	1 (2.9%)	0.2486
premature ventricular contraction	1 (1.2%)	0 (0.0%)	1 (2.9%)	0.2486

Mild adverse events were defined as complications resolving spontaneously within one hour and included rash (14 cases, 17.3%), pruritus (5 cases, 6.2%), nausea (9 cases, 11.1%) and headache (3 cases, 3.7%). In 11 out of 14 cases rashes were reported in the rhTNFR-Fc group. Moderate adverse events were defined as complications that may be immediately alleviated by medical interventions and included skin allergies (16 cases, 19.8%), fever (2 cases, 2.5%), palpitations (12 cases, 14.8%), dyspnea (2 cases, 2.5%), chest pain (1 case, 1.2%), abdominal pain (4 cases, 4.9%) and hypertension (9 cases, 11.1%). Severe adverse events were defined as fatal or requiring inpatient hospitalization and included papilledema (2 cases, 2.5%), laryngeal edema (1 case, 1.2%) and premature ventricular contraction (1 case, 1.2%).

### Long-term adverse events

Long-term follow-up data was available for 172 AS patients, including 87 patients receiving 200mg Infliximab every six weeks and 85 patients receiving 25 mg rhTNFR-Fc twice a week for 104 weeks of follow-up. Patient enrollment started in 2008 and follow-up was concluded in 2013. This study observed a 57% (230/402) loss to follow-up. Loss to follow-up was due to the financial burden associated with anti-TNF-α therapy (considering these therapies are often not fully reimbursed by health insurance) in 162 patients which were subsequently treated with NSAID/DMARD co-therapy, 20 patients discontinued due to treatment failure and were subsequently treated with NSAID/DMARD co-therapy, 19 patients discontinued treatment due to adverse events and were subsequently treated with NSAID/DMARD co-therapy, 23 patients relocated and were lost to follow-up, 5 patients were lost to follow-up for unknown reasons, 1 patient discontinued treatment due to non-small cell lung cancer 13 weeks after start of infliximab therapy.

Long-term adverse events occurred in 59 patients (34.3%), including 19/85 patients (19/172; 11.0%) treated with rhTNFR-Fc and 40/87 patients (40/172; 23.3%) treated with Infliximab, constituting a significant difference (p = 0.0013). See Table 3. None of the patients experiencing adverse events at long-term follow-up experienced adverse events at short-term follow-up. Patients experiencing adverse events were younger (37.7 ± 20.2 versus 42.3 ± 19.6 years, p = 0.1461) and had shorter disease duration (6.29 ± 6.45 versus 10.87 ± 9.51, p = 0.0011). Additionally, patients experiencing an adverse event had higher baseline values of CRP (44.8 ± 18.7 versus 35.7 ± 19.6 mg/dl, p = 0.0039) and ESR (42.9 ± 16.5 versus 36.9 ± 18.0 mg/dl, p = 0.0345).

**Table 3:** Baseline characteristics of patients experiencing long-term adverse events

	All patients	Non-adverse event patients	Adverse event patients	P-value
Demographics	(n = 172)	(n = 113)	(n = 59)	
Age (years)	40.6 ± 18.5	42.3 ± 19.6	37.7 ± 20.2	0.1461
Male (%)	98.8%	98.2%	100%	9.39E-17
duration (years)	9.8 ± 8.5	10.9 ± 9.5	6.3 ± 6.5	0.0011
Type				
Axial phenotype (%)	98.3%	99.1%	98.3%	0.6381
Biologic assessment				
HLA-B27, no (%)	100%	100%	100%	
Serum CRP (mg/dl)	39.6 ± 17.3	35.7 ± 19.6	44.8 ± 18.7	0.0039
ESR (mg/dl)	38.5 ± 18.7	36.9 ± 18.0	42.9 ± 16.5	0.0345
Drug treatment				
Infliximab (%)	50.6% (87)	41.6% (47)	67.8% (40)	0.0013
rhTNFR-Fc (%)	49.4% (85)	58.4% (66)	32.2% (19)	

CRP = C-reactive protein. ESR = erythrocyte sedimentation rate. Axial phenotype is ankylosing spondylitis which initially predominantly affects the spine and pelvic joints.

Patients commonly reported infectious diseases as adverse events, including pneumonia, urinary tract infection, otitis media, tuberculosis, abscess and oral candidiasis. See Table 4. Specifically, pneumonias (13 cases, 22.0%) and urinary tract infections (17 cases, 28.9%) combined constituted half of all adverse events. While there was no significant difference between the two treatment groups with regards to the incidence of pneumonias, urinary tracts occurred more frequently in the Infliximab-group (2 versus 15, p = 0.0325).

**Table 4:** Comparison of long-term adverse events.

	All adverse event patients	rhTNFR-Fc	Infliximab	P-value
	(n = 59)	(n = 19)	(n = 40)	
**Infectious disease (%)**				
Pneumonia	13 (22.0%)	5 (26.3%)	8 (20.0%)	0.5844
Urinary tract infection	17 (28.8%)	2 (10.5%)	15 (37.5%)	0.0325
Otitis media	8 (13.6%)	1 (5.3%)	7 (17.5%)	0.1995
Tuberculosis	6 (10.2%)	4 (21.1%)	2 (5.0%)	0.0566
Abscess	2 (3.4%)	0 (0%)	2 (5.0%)	0.3213
Oral candidiasis	1 (1.7%)	0 (0%)	1 (2.5%)	0.4869
**The other AEs (%)**				
Elevation of transaminase	3 (5.1%)	2 (10.5%)	1 (2.5%)	0.1897
Anemia	2 (3.4%)	1 (5.3%)	1 (2.5%)	0.5836
Hematuresis	1 (1.7%)	0 (0%)	1 (2.5%)	0.4869
Constipation	4 (6.8%)	4 (21.1%)	0 (0%)	0.0026
Weight loss	1 (1.7%)	0 (0%)	1 (2.5%)	0.4869
Exfoliative dermatitis	1 (1.7%)	0 (0%)	1 (2.5%)	0.4869

## Discussion

In China, anti-TNF-α therapy is widely applied as treatment for various rheumatic diseases due to its clinical efficacy [15–16]. However, since their clinical availability in 2006, studies have reported adverse events inconsistent with original drug-safety profiles [17], and showed anti-TNF-α treatment can result in serious adverse events [18–19]. Nagy et al reported 3.4% (11/324) patients experiencing adverse events associated with infliximab treatment, 7 (7/324, 2.2%) of which serious enough to discontinue treatment [17]. Similarly, Lee et al noted 23.3% (35/150) of patients with various rheumatic diseases suffering cutaneous side effects associated with infliximab, adalimumab and etanercept therapy [20]. Bonafede et al reported on the treatment adherence of patients receiving infliximab treatment for AS and reported 30% (14/46) of patients discontinued treatment of infliximab completely within 1 year, whereas 57% (26/46) completed a 1 year treatment regimen [21]. Huang et al reported up to 65% prevalence of adverse events in AS patients receiving treatment with biological [22]. In our own study, according to Chinese prescription protocols, we administered lower dosages of the respective TNF-α blockers than reported in the literature, which may in turn be associated with milder adverse event profile and better tolerability. As use becomes more widespread, owing to anti-TNF-α therapy becoming more established as the first-line of treatment, knowledge of the adverse events associated with anti-TNF-α treatment is critical in providing care. As such, to our knowledge, we provide the first report on the adverse events associated with long-term mono-treatment of rhTNFR-Fc and Infliximab in Chinese Han patients with ankylosing spondylitis.

Our study showed a majority (46/81, 56.8%) of the short-term adverse events were considered moderate, with approximately 11.4% (46/402) undergoing a short-term moderate adverse event. These outcomes correlate with literature where incidence rates of 0.59% (21/3555) and 13.2% were reported with infliximab infusion [23–25]. While severe adverse events only accounted for <1% (4/402) in our study, these were generally fatal, indicating the importance of monitored administration in centers capable of managing medical emergencies. Most adverse events in our study reported during long-term treatment were infectious diseases, a finding that is consistent with literature [26, 27]. Even if patients initially respond favorably to treatment vigilance is required as patients may still suffer adverse events at long-term follow-up. For example, none of the 59 patients suffering adverse events at long-term follow-up had side effects at short-term treatment. Thus, comprehensive knowledge of possibly occurring adverse events associated with long-term treatment with TNF-α blockers is critical [28].

Understanding which patients are predisposed to adverse events following long-term anti-TNF-α therapy may help avoiding such adverse events in the future. Our study showed an increased likelihood of experiencing adverse events in both short-term and long-term treatment in patients who had shorter disease duration. The brevity of their disease duration may be a derivative of the intensity of their disease and thus also the general state of health. Also, patients experiencing adverse events within 2 hours of start of therapy were more likely to suffer from peripheral joint symptoms than axial skeleton complaints. Finally, serum CRP and ESR were found to be predictive of the occurrence of adverse events. At short-term treatment these biomarkers were lower than in patients who did not experience adverse events, but still significantly elevated compared to the general public. At long-term treatment these blood biomarkers were elevated above both the non-adverse events patients and the general public. This difference may be explained by the capability of CRP to react with nuclear antigen, and promote the phagocytosis of the complex by macrophages [29]. Additionally, by complexing with FcTR, CRP can speed up the clearance of auto-antigens and thus prevent auto immune responses [30]. Therefore, a relatively lower CRP might be a sign of dysregulation of the immune system.

Our study has several limitations to the extent to which the results can be generalized. First of all, the ranges of age and disease duration of our selected patients were appreciably wide, alluding to a heterogeneous population. Also, we encountered a large loss to follow-up due to the economic burden of these costly drugs. Lastly, the BASDAI (Bath Ankylosing Spondylitis Disease Activity Index) was not employed, impeding us in evaluating the correlation between disease activity and the prevalence of adverse events [31].

## Conclusion

Anti-TNF-α therapy using rhTNFR-Fc and Infliximab can cause both short-term and long-term adverse events. Even though the majority of such adverse events are treatable, vigilance and detailed knowledge of possibly occurring adverse events is required in order to prevent unnecessary morbidity. Assessing baseline characteristics may help isolate at-risk patients, offering them alternative treatment options. We found predominantly peripheral skeletal disease involvement and blood biomarker levels to be predictive of the prevalence of adverse events.
