# Correction: Adverse Events of Anti-Tumor Necrosis Factor α Therapy in Ankylosing Spondylitis

**DOI:** 10.1371/journal.pone.0127488

**Published:** 2015-04-27

**Authors:** Qiang Tong, Qing Cai, Tristan de Mooij, Xia Xu, Shengming Dai, Wenchun Qu, Dongbao Zhao

In the Results section of the Abstract, there are errors in the percentages and the ratios for both the chest pain and the tuberculosis data. The correct values for chest pain are (0.2%; 1/402), and the correct values for tuberculosis are (3.5%; 6/172).
